# Clinical Relevance of a Vancomycin 24 h Area under the Concentration—Time Curve Values Using Different Renal Function Equations in Bayesian Dosing Software

**DOI:** 10.3390/jpm13010120

**Published:** 2023-01-05

**Authors:** Hyun-Ki Kim, Tae-Dong Jeong

**Affiliations:** 1 Department of Laboratory Medicine, University of Ulsan College of Medicine, Ulsan University Hospital, Ulsan 44033, Republic of Korea; 2 Department of Laboratory Medicine, Ewha Womans University College of Medicine, Seoul 07804, Republic of Korea

**Keywords:** estimated glomerular filtration rate, therapeutic drug monitoring, vancomycin

## Abstract

With the updated 2020 vancomycin therapeutic drug monitoring (TDM) guidelines suggesting a ratio of area under the curve over 24 h to a minimum inhibitory concentration (AUC_24_/MIC) as a target from the Infectious Diseases Society of America, an accurate estimation of AUC_24_ has become more critical. We aim to compare the AUC_24_ using Bayesian dosing software according to various estimated glomerular filtration rate (eGFR) equations in order to analyze the clinical impact of eGFR in vancomycin TDM. We reviewed the TDM dataset of 214 adult patients and analyzed the AUC_24_ values from various renal function equations, including the Cockcroft-Gault (C-G), the modification of diet in renal disease (MDRD), the chronic kidney disease epidemiology collaboration (CKD-EPI), and the revised Lund–Malmö. The AUC_24_/MIC results (assuming a MIC of 1 mg/L) were divided into three groups as follows: <400, 400–600, and >600. Additionally, we compared the group agreement between the C-G and the three eGFR formulas. Although there was a statistically significant difference in the AUC24 of the MDRD and the CKD-EPI formulas compared to the C-G, the group concordance rate of the eGFR formula was 95.2–100%, which indicates no clinical significance. The clinical impact of the eGFR formula type on drug dosing recommendations in vancomycin TDM using Bayesian software was insignificant in clinical practice.

## 1. Introduction

Vancomycin is a drug of choice for treating methicillin-resistant *Staphylococcus aureus* (MRSA) infections [1]. The latest international guidelines for vancomycin therapeutic drug monitoring (TDM) recommend administering 15–20 mg/kg of vancomycin every 8–12 h for severe MRSA infections [2]. However, because vancomycin has a narrow therapeutic index with large inter- and intra-individual variability in pharmacokinetic (PK) parameters, its treatment effect should be monitored through continuous TDM [2]. Trough concentration (C_trough_)-guided TDM within 15–20 ug/mL was recommended in the past [3]. However, today, drug dosing is recommended based on a ratio of the area under the curve over 24 h to the minimum inhibitory concentration (AUC_24_/MIC) [2]. Because vancomycin is mainly eliminated through the kidneys, the concentration of vancomycin in the blood and the drug’s therapeutic effect are closely related to renal function [4]. Therefore, a renal function estimate is usually included in the population PK model as a covariate [5].

A representative index used to evaluate renal function in clinical practice is the glomerular filtration rate (GFR) [6]. In clinical practice, we calculate the estimated GFR (eGFR) using either creatinine, cystatin C, or both. Historically, the Cockcroft-Gault (C-G) equation is the most widely used equation for calculating creatinine clearance, and more recently, the Modification of Diet in Renal Disease (MDRD) and Chronic Kidney Disease Epidemiology Collaboration (CKD-EPI), which can estimate eGFR more accurately than C-G, have been introduced and validated [7,8,9,10,11]. Since the C-G formula calculates the creatinine clearance, the unit is mL/min, and the MDRD and CKD-EPI use the unit of mL/min/1.73 m^2^, corrected for body surface area (BSA). As the MDRD or CKD-EPI performs better than the C-G in estimating GFR, some researchers have proposed using a more accurate eGFR formula in the TDM area [12]. Still, there has yet to be an international consensus on the eGFR equation for TDM.

Vancomycin TDM is commonly utilized for appropriate drug administration in clinical practice. Usually, vancomycin PK analysis uses commercialized Bayesian dosing software, and different laboratories use different eGFR equations. In vancomycin TDM using Bayesian dosing software, studies on the effect of the eGFR formula on AUC_24_/MIC and drug dosing recommendations are lacking. Therefore, we aim to compare the AUC_24_/MIC using a Bayesian dosing software depending on the eGFR formula in order to analyze the impact of the vancomycin drug dosing in clinical practice.

## 2. Materials and Methods

### 2.1. Study Subjects and Data Collection

**Figure 1:**
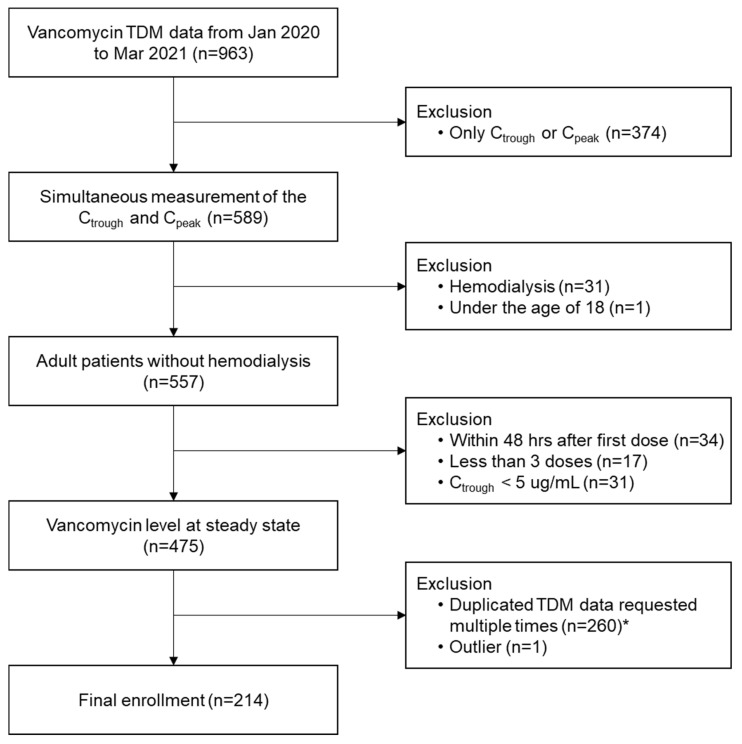
Schematic diagram of the data collection process. * In cases where TDM was requested multiple times for the same patient, the first set of data was selected. Abbreviations: C_trough_, trough concentration of vancomycin; C_peak_, peak concentration of vancomycin; and TDM, therapeutic drug monitoring.

We collected TDM data through a retrospective medical record review. From January 2020 to March 2021, 963 cases of vancomycin TDM in 405 patients were recorded at Ewha Womans University Seoul Hospital, Seoul, Korea. Of these, 589 cases measured a drug concentration of vancomycin in blood at two time points (trough and peak), and cases excluded patients under 18 years of age (*n* = 1) and patients with HD (*n* = 31). To analyze cases where the vancomycin blood concentration reached a steady state, cases where less than 48 h had elapsed after the first vancomycin administration (*n* = 34), three or fewer drug administrations (*n* = 17), and a C_trough_ of less than 5.0 ug/mL (*n* = 31) were excluded. When multiple requests for vancomycin TDM were received from the same patient, we chose the first TDM data. A total of 214 patients were finally enrolled after excluding one outlier (Figure 1). In one outlier case, the sampling time for the drug concentration measurement described in the TDM request form did not match the actual drug administration time.

### 2.2. Serum Vancomycin Concentration Measurements

Venous blood was drawn within 30 min of the next drug dose (C_trough_) and 1 h after the intravenous dose (C_peak_). The vancomycin concentration was measured using the Architect i1000 SR analyzer (Abbott, Wiesbaden, Germany). During the study period, we performed the internal quality control of vancomycin with three concentrations of quality control materials. The within-laboratory imprecision was 3.2% at low concentration, 2.0% at medium concentration, and 2.3% at high concentration. In addition, we participated in proficiency testing (PT) for vancomycin conducted by the Korean Association of External Quality Assessment Service, and all the PT results were acceptable.

### 2.3. TDM Analysis Tool and Calculation of eGFR

For vancomycin TDM analysis, we used the MwPharm++ (Mediware, Praha, Czech Republic) program [13,14]. This software was able to apply various types of eGFR calculations. In this study, we performed vancomycin TDM analysis according to four types of eGFR formulas, including C-G [7], MDRD [15], CKD-EPI [15], and revised Lund–Malmö (LM) [16]. Briefly, the vancomycin TDM analysis procedure was as follows. The data, such as the patient’s age, sex, serum creatinine concentration, vancomycin drug administration information, and blood vancomycin drug concentration information, were entered into the software, and then Bayesian fitting was performed. As a result of the analysis, we obtained AUC_24_/MIC values according to each eGFR formula. In this study, we assume a MIC of 1 mg/L [2].

### 2.4. Statistical Analysis

The AUC_24_/MIC results calculated by each eGFR formula were divided into three groups (subtherapeutic, <400; therapeutic, 400–600; and toxic, >600) as recommended by international guidelines [2], and we compared the agreement between the groups of the C-G formula and the three eGFR formulas. Additionally, to analyze the difference according to the creatinine concentration, the serum creatinine concentration of the study group was divided into quartiles and compared by subgroup. Based on the C-G formula, we compared the AUC_24_ of the three eGFR formulas using the Wilcoxon signed-rank test.

According to a normal distribution, continuous variables are expressed as the mean ± standard deviation or median (first quartile, Q1; third quartile, Q3). Statistical analysis was performed using R version 4.0.4 (R Foundation for Statistical Computing, Vienna, Austria) and Analyse-it for Microsoft Excel 5.92 (Analyse-it Software Ltd., Leeds, UK). A *p* value of less than 0.05 was considered statistically significant.

## 3. Results

### 3.1. Characteristics of Study Subjects

A total of 214 subjects were enrolled. Males comprised 60% of the study population, and the mean age was 72 years. The median serum creatinine concentration was 0.61 mg/dL (53.9 μmol/L; 1 mg/dL = 88.4 μmol/L), and the median C_trough_ and C_peak_ values were 11.6 μg/mL and 28.8 μg/mL, respectively. The median daily vancomycin dose was 29.7 mg/kg. Table 1 describes the characteristics of the patients in the study.

### 3.2. AUC_24_ According to the eGFR Formula

The median values of AUC_24_ according to each eGFR formula in all study subjects were 441.9 mg∙h/L for C-G, 437.4 mg∙h/L for MDRD, 440.3 mg∙h/L for CKD-EPI, and 444.5 mg∙h/L for the revised LM. Compared to the C-G, the median difference (95% CI) of the MDRD was −3.1 (−3.4, −2.9; *p* < 0.001), and that of the CKD-EPI was −1.1 (−1.4, −0.8; *p* < 0.001). On the other hand, the AUC_24_/MIC of the revised LM was not significantly different from C-G. A similar pattern was observed in the analysis of the creatinine concentration quartile groups (Table 2).

**Figure 2:**
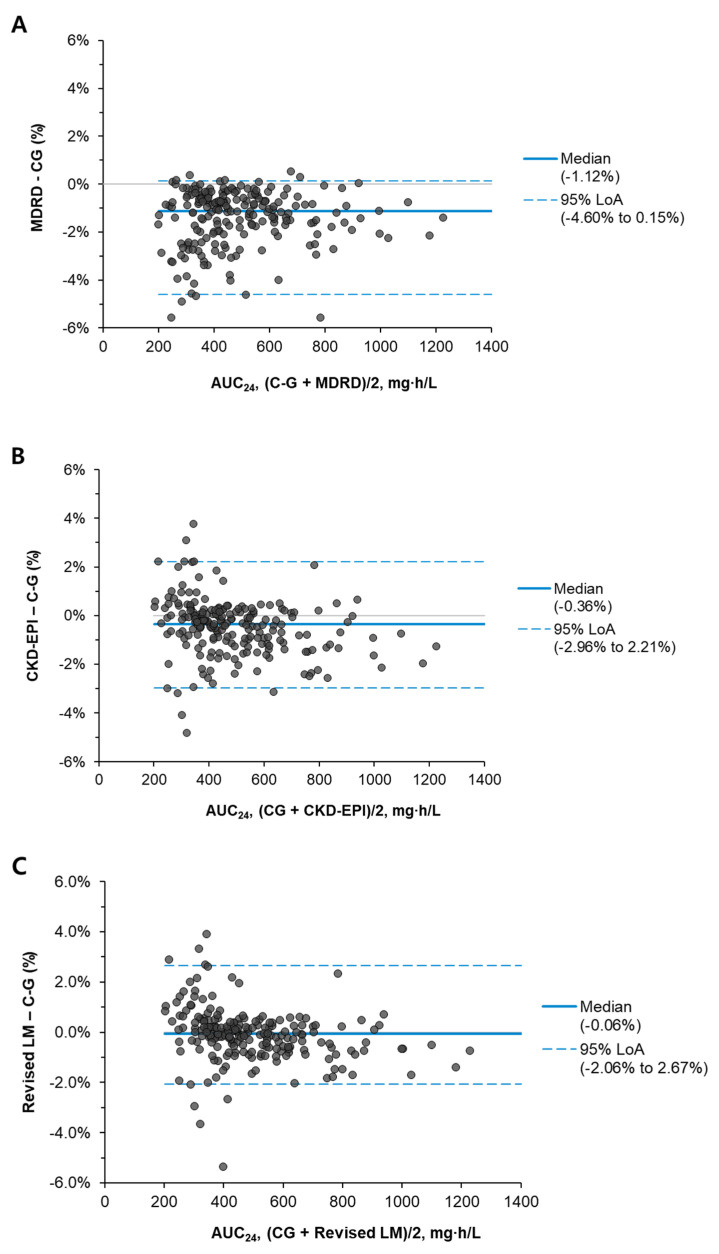
Bland–Altman plots for AUC_24_ of vancomycin according to the estimated glomerular filtration rate equations. (**A**) Between C-G and MDRD. (**B**) Between C-G and CKD-EPI. (**C**) Between C-G and the revised LM. Outliers were excluded ((**A**), *n* = 2; (**B**), *n* = 2; and (**C**), *n* = 3). See Appendix A (Figure A1) for all data results, including outliers. Abbreviations: AUC, area under the curve over 24 h; C-G, Cockcroft-Gault; CKD-EPI, Chronic Kidney Disease Epidemiology Collaboration; MDRD, Modification of Diet in Renal Disease; LoA, limit of agreement; and LM, Lund–Malmö.

Regarding the AUC_24_/MIC interval agreement based on the C-G formula, the weighted kappa value was 0.972 for MDRD, 0.989 for CKD-EPI, and 0.983 for the revised LM. The group concordance of the eGFR formula was 100% in the AUC_24_/MIC < 400 group and 98.1% in the AUC_24_/MIC > 600 group, showing no difference between the eGFR formulas. In the AUC_24_/MIC 400–600 group, the group concordance compared with the C-G formula was highest for CKD-EPI at 98.8%, followed by revised LM at 97.6%, and MDRD at 95.2% (Table 3). Figure 2 shows the Bland–Altman plot of each eGFR formula for AUC_24_/MIC.

## 4. Discussion

We evaluated the effect of the eGFR formulas on the AUC24/MIC of vancomycin TDM using Bayesian drug analysis software, MwPharm++. There was a statistically significant difference in the AUC_24_ of the MDRD and CKD-EPI compared to the C-G. However, the effect of these differences on actual vancomycin drug dosing in clinical practice would be insignificant. The AUC_24_ of MDRD and CKD-EPI showed a median difference of −3.1% and −1.1%, respectively, compared to the AUC_24_ of C-G in 214 patients. This difference means that there is no clinically significant difference in the change in the drug dose administered to the patient. According to international guidelines for vancomycin TDM, the AUC_24_/MIC corresponding to the optimal therapeutic effect is 400–600 [2]. If the vancomycin AUC_24_/MIC is less than 400, we should increase the drug dose, and if it is more than 600, we should reduce the dose. Based on C-G, our results show that the group agreement of the AUC_24_/MIC interval was 97.7% for MDRD, 99.1% for CKD-EPI, and 98.6% for the revised LM. Therefore, no matter which eGFR formula we select in the MwPharm++ software, there will be no significant difference in the recommended drug dose for patients in clinical practice.

The GFR is the flow rate of plasma passing through the glomerular membrane per minute. Since the patient’s GFR cannot be directly measured in clinical practice, it is evaluated in two ways: GFR is measured indirectly using an exogenous substance, or GFR is estimated using an endogenous substance. Exogenous substances used for GFR measurement include non-radioactive substances such as inulin and iohexol and radioactive substances such as ^51^Cr-ethylenediaminetetraacetic acid, ^99m^Tc-diethylenetriaminepentaacetic acid, and ^125^I-iothalamate. Measuring the urine clearance of inulin after continuous inulin infusion is considered the gold standard for measuring GFR. However, this method is inconvenient because inulin should be continuously injected intravenously into the patient, urine samples should be collected several times to calculate the clearance, and even catheterization may be required to evaluate the exact amount of urine. In addition, the measurement cost is high, time-consuming, and labor-intensive for the assay, limiting its universal use in clinical laboratories. For this reason, the eGFR is calculated in clinical practice by measuring the serum concentration of representative endogenous markers, such as creatinine, cystatin C, or both.

Many clinical laboratories using laboratory information systems automatically calculate and report eGFR [17]. The 2012 Kidney Disease: Improving Global Outcomes clinical practice guidelines for CKD recommend using the CKD-EPI to calculate eGFR for adults unless an alternative creatinine-based GFR estimating equation is acceptable [15]. However, various eGFR equations are used in clinical practice. For example, eGFR formulas used by clinical laboratories participating in the 2017 College of American Pathologists general chemistry proficiency testing survey included C-G (3%), isotope dilution mass spectrometry (IDMS) non-traceable MDRD (16%), IDMS traceable MDRD (53%), CKD-EPI (25%), etc. [17]. Of course, calculating eGFR from creatinine concentration in clinical laboratories may differ from the formula for eGFR used in TDM analysis. To date, no internationally agreed single eGFR formula is recommended for TDM analysis.

Although the eGFR values calculated by each eGFR formula were different from each other, the clinical effect of the eGFR formula type on the vancomycin AUC_24_/MIC in the MwPharm++ software was not significant. A Bayesian method estimates parameters specific to the patient using both prior information and measured values for the patient; the effect of differences in the renal function estimates might therefore be diluted [18]. Nevertheless, since more accurate GFR estimation will help with more accurate vancomycin clearance estimation and drug concentration prediction in Bayesian dosing software, efforts to apply a better renal function estimation formula should continue. So far, most of the vancomycin population PK parameters used in a Bayesian method, including those built in the MwPharm++ program, are set based on C-G [5]. Although some researchers have reported studies on PK parameters based on the 2009 CKD-EPI equation [19,20], as other new formulas such as the 2021 CKD-EPI equation have been proposed [21], it is necessary to develop upon the more recent eGFR formula with improved accuracy for TDM analysis programs in the future.

This study had some limitations. First, we used only one TDM analysis program. Since TDM analysis software usually uses PK parameters based on the C-G formula and commonly applies Bayesian analysis techniques, other TDM analysis programs may produce similar results. Second, the blood drug concentration and blood collection time described in the TDM test request were analyzed, but there is the possibility that this information needed to be more accurate. Determining the exact timing of blood sampling for TDM analysis is still challenging in clinical practice. Third, we assumed a MIC of 1 mg/L.

## 5. Conclusions

The effect of the eGFR formula type on drug dosing recommendations in vancomycin TDM using a Bayesian analysis technique was insignificant. Since a more accurate GFR estimation can increase the accuracy of TDM analysis, a population PK parameter based on the eGFR formula with better accuracy than C-G, such as the CKD-EPI, needs to be applied to the TDM analysis programs in clinical practice.

## Figures and Tables

**Table 1:** Characteristics of the study population.

Characteristic	Value
Total number, *n*	214
Male gender, *n* (%)	129 (60.3)
Age, years (median [Q1, Q3])	72 (60, 79)
Body weight, kg (mean ± SD)	59.8 ± 13.2
Height, cm (mean ± SD)	162.9 ± 8.9
BSA, m^2^ (median, [Q1, Q3])	1.62 (1.50, 1.78)
BMI, kg/m^2^ (mean ± SD)	22.5 ± 4.4
Serum creatinine, mg/dL (median [Q1, Q3])	0.61 (0.47, 0.81)
eGFR, mL/min *	
C-G (median [Q1, Q3])	80.6 (54.3, 113.7)
MDRD (median [Q1, Q3])	112.7 (78.8, 154.0)
CKD-EPI (median [Q1, Q3])	91.2 (72.3, 107.2)
Revised LM (median [Q1, Q3])	82.8 (65.2, 99.7)
Measured vancomycin C_trough_, μg/mL (median [Q1, Q3])	11.6 (8.1, 16.4)
Measured vancomycin C_peak_, μg/mL (median [Q1, Q3])	28.8 (25.0, 37.0)
Daily vancomycin dose, mg/kg (median [Q1, Q3])	29.7 (25.0, 37.0)

Continuous variables are expressed as mean ± SD or median (Q1, Q3) according to data distribution. * eGFR with BSA normalization removed as follows: $GFR\;\left(\mathrm{mL}/\min\right)=GFR\left(\mathrm{mL}/\min/1.73\;m^{2}\right)\times BSA/1.73$. Abbreviations: BMI, body mass index; BSA, body surface area (calculated by Du Bios formula, $BSA=0.007184\times Body\;weight\;in\;kg^{0.425}\times Height\;in\;cm^{0.725}$; C-G, Cockcroft-Gault; CKD-EPI, Chronic Kidney Disease Epidemiology Collaboration; eGFR, estimated glomerular filtration rate; C_trough_, trough concentration; C_peak_, peak concentration; LM, Lund–Malmö; MDRD, Modification of Diet in Renal Disease; Q1, first quartile; Q3, third quartile; and SD, standard deviation.3.2. AUC_24_ According to the eGFR Formula.

**Table 2:** Predictive performance of therapeutic drug monitoring of vancomycin depending on the eGFR equation.

Variable	eGFR Equation			
	C-G	MDRD	CKD-EPI	Revised LM
All				
Median AUC_24_ (95% CI)	441.9 (420.1, 468.5)	437.4 (415.7, 466.2)	440.3 (418.9, 468.6)	444.5 (422.6, 475.1)
Median difference, % (95% CI)	Reference	−3.1 (−3.4, −2.9) ^b^	−1.1 (−1.4, −0.8) ^b^	−0.2 (−0.5, 0.1) ^c^
Creatinine 0.16–0.47 mg/dL * (Q1)				
Median AUC_24_ (95% CI)	393.4 (355.8, 433.3)	388.8 (350.1, 424.0)	392.4 (355.8, 432.5)	392.6 (356.1, 433.7)
Median difference, % (95% CI)	Reference	−5.6 (−6.9, −4.4) ^b^	1.7 (0.8, 2.8) ^b^	2.3 (1.4, 3.4) ^b^
Creatinine 0.48–0.61 mg/dL * (Q2)				
Median AUC_24_ (95% CI)	419.9 (363.0, 456.1)	414.7 (354.8, 448.9)	418.0 (360.1, 455.2)	424.7 (362.2, 464.5)
Median difference, % (95% CI)	Reference	−6.1 (−8.0, −4.5) ^b^	−1.3 (−2.4, −0.5) ^b^	0.1 (−0.8, 0.7) ^c^
Creatinine 0.62–0.81 mg/dL * (Q3)				
Median AUC_24_ (95% CI)	452.4 (412.2, 545.8)	447.8 (406.4, 541.1)	450.5 (406.5, 540.8)	451.7 (408.8, 542.8)
Median difference, % (95% CI)	Reference	−5.8 (−7.4, −4.1) ^b^	−4.0 (−5.6, −2.7) ^b^	−1.7 (−2.7, −0.6) ^a^
Creatinine 0.82–2.11 mg/dL * (Q4)				
Median AUC_24_ (95% CI)	562.8 (509.4, 630.2)	559.0 (504.0, 616.2)	558.3 (503.7, 619.7)	559.7 (506.2, 626.8)
Median difference, % (95% CI)	Reference	−7.1 (−9.3, −5.3) ^b^	−7.0 (−9.0, −5.4) ^b^	−4.2 (−5.7, −3.0) ^b^

^a^, *p* < 0.05; ^b^, *p* < 0.001; and ^c^, non-significant. Comparing the C-G formula and each eGFR formula using the Wilcoxon signed-rank test. * For conversion from mg/dL to μmol/L, ×88.4. Abbreviations: C-G, Cockcroft-Gault; CI, confidence interval; CKD-EPI, Chronic Kidney Disease Epidemiology Collaboration; eGFR, estimated glomerular filtration rate; MDRD, Modification of Diet in Renal Disease; Q, quartile; and LM, Lund–Malmö.

**Table 3:** Agreement between Cockcroft-Gault and the other eGFR equations for prediction of AUC_24_/MIC.

eGFR Equation	AUC_24_/MIC	AUC_24_/MIC by C-G			Weighted Kappa (95% CI)
		<400	400–600	>600	
MDRD	<400	79	4	0	0.972 (0.948, 0.996)
	400–600	0	79	1	
	>600	0	0	51	
CKD-EPI	<400	79	1	0	0.989 (0.973, 1.000)
	400–600	0	82	1	
	>600	0	0	51	
Revised LM	<400	79	2	0	0.983 (0.964, 1.000)
	400–600	0	81	1	
	>600	0	0	51	

Abbreviations: AUC_24_/MIC (assuming a MIC of 1 mg/L), a ratio of the area under the curve over 24 h to the minimum inhibitory concentration; C-G, Cockcroft-Gault; CI, confidence interval; CKD-EPI, Chronic Kidney Disease Epidemiology Collaboration; eGFR, estimated glomerular filtration rate; MDRD, Modification of Diet in Renal Disease; and revised LM, revised Lund–Malmö.

**Figure A1:**
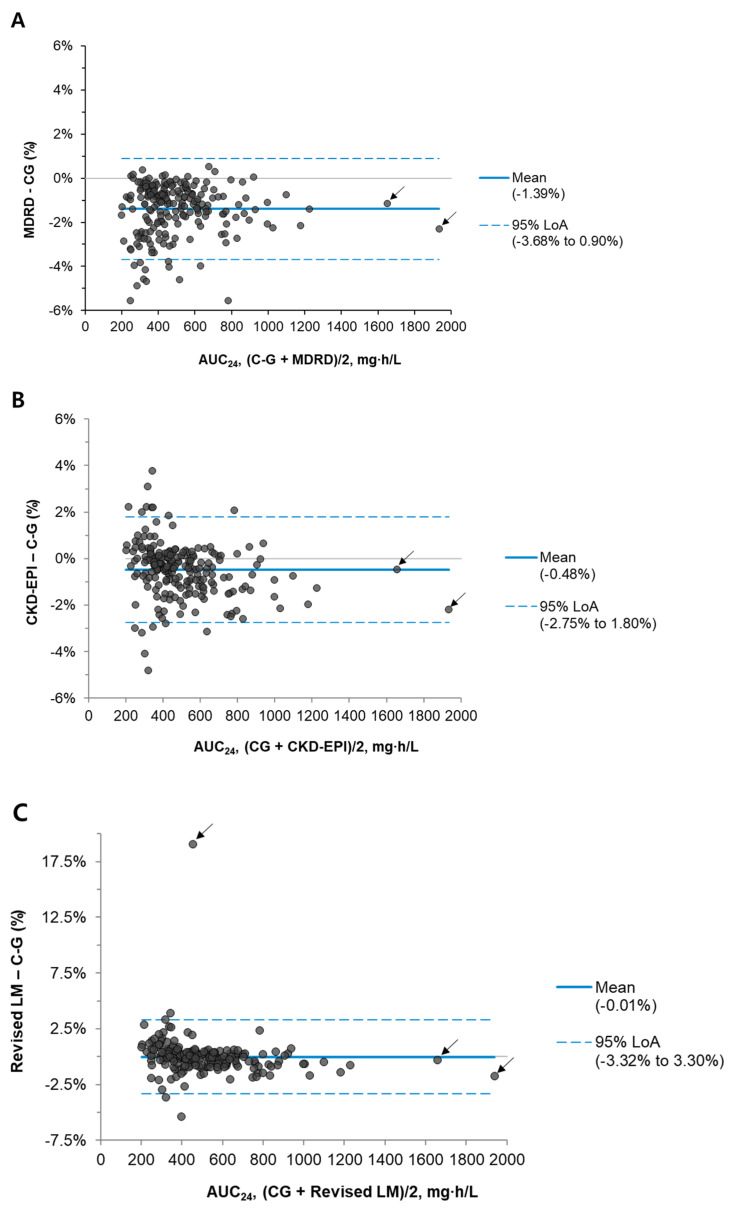
Bland–Altman plots for AUC_24_ of vancomycin according to estimated glomerular filtration rate equations. (**A**) Between C-G and MDRD. (**B**) Between C-G and CKD-EPI. (**C**) Between C-G and revised LM. Arrows indicate outliers. Abbreviations: AUC, area under the curve over 24 h; C-G, Cockcroft-Gault; CKD-EPI, Chronic Kidney Disease Epidemiology Collaboration; MDRD, Modification of Diet in Renal Disease; LoA, limit of agreement; and LM, Lund–Malmö.

## Data Availability

The data are not publicly available due to ethnic restrictions.
